# Dynamic improvement of tricuspid valve leaflets coaptation and tricuspid regurgitation without surgical treatment: a case report

**DOI:** 10.1093/ehjcr/ytaf552

**Published:** 2025-10-23

**Authors:** Haruka Minami, Ayano Yoshida, Kyohei Onishi, Kosuke Fujita, Gaku Nakazawa

**Affiliations:** Division of Cardiology, Department of Medicine, Kindai University Faculty of Medicine, 1-14-1, Mihara-dai, Minami-ku, Sakai, 590-0197, Japan; Division of Cardiology, Department of Medicine, Kindai University Faculty of Medicine, 1-14-1, Mihara-dai, Minami-ku, Sakai, 590-0197, Japan; Division of Cardiology, Department of Medicine, Kindai University Faculty of Medicine, 1-14-1, Mihara-dai, Minami-ku, Sakai, 590-0197, Japan; Division of Cardiology, Department of Medicine, Kindai University Faculty of Medicine, 1-14-1, Mihara-dai, Minami-ku, Sakai, 590-0197, Japan; Division of Cardiology, Department of Medicine, Kindai University Faculty of Medicine, 1-14-1, Mihara-dai, Minami-ku, Sakai, 590-0197, Japan

**Keywords:** tricuspid regurgitation, Heart failure with reduced ejection fraction, Ischaemic heart disease, Low-flow-low-gradient severe aortic stenosis, Atrial flutter, Case report

## Abstract

**Background:**

Tricuspid regurgitation is a prevalent condition among patients with heart failure. Functional tricuspid regurgitation arises from multiple contributing factors, and tricuspid regurgitation severity is sensitive to changes in volume status. Importantly, significant tricuspid regurgitation is associated with adverse outcomes; therefore, tricuspid regurgitation management is crucial.

**Case summary:**

We describe the case of a 92-year-old man was diagnosed with decompensated heart failure with underlying reduced ejection fraction, multivessel coronary artery disease, low-flow low-gradient aortic stenosis, atrial flutter, and severe tricuspid regurgitation without leaflet coaptation. Owing to the high surgical risk, a minimally invasive treatment strategy was adopted. The patient underwent percutaneous coronary intervention of the left coronary artery, including the left main coronary artery, after which sinus rhythm was spontaneously restored. However, the severity of tricuspid regurgitation remained unchanged posttreatment. Thus, transcatheter aortic valve implantation was subsequently performed. Remarkably, echocardiography on the day after transcatheter aortic valve implantation revealed an improvement in tricuspid regurgitation severity from severe to moderate, with leaflet coaptation and size reduction of the right atrium and right ventricle.

**Discussion:**

This case highlights that severe tricuspid regurgitation, even without leaflet coaptation, can be improved through comprehensive management of ischaemia, valvular heart disease, and arrhythmia in the absence of surgical treatment. This treatment approach may offer clinical benefits to patients at high surgical risk.

Learning points

- Significant tricuspid regurgitation (TR), even without leaflet coaptation, can be improved through a comprehensive nonsurgical treatment strategy for myocardial ischaemia, aortic stenosis, and atrial arrhythmias.
- This case showed the possibility of transcatheter therapies such as PCI and TAVI, along with rhythm control, to achieve right heart reverse remodelling and improve TR severity even in high-surgical-risk patients, and leading to better clinical outcome.

## Introduction

Functional tricuspid regurgitation (TR) is a common comorbidity in patients with advanced heart failure (HF),^1,2^ and significant TR is associated with adverse outcomes.^3^ Management of TR is often complicated, especially in older or high-risk patients who are not candidates for surgical intervention. Although transcatheter approaches for left-sided valvular disease and coronary artery disease are well-established modalities, their impact on severe functional TR is not fully understood. We describe here a case of marked improvement in TR without leaflet coaptation following coronary intervention, transcatheter aortic valve implantation (TAVI), sinus rhythm restoration.

## Summary figure

AS, aortic stenosis; LCA, left coronary artery; LFLG, low-flow low-gradient; PCI, percutaneous coronary intervention; TAVI, transcatheter aortic valve implantation; TR, tricuspid regurgitation.

**Figure:**
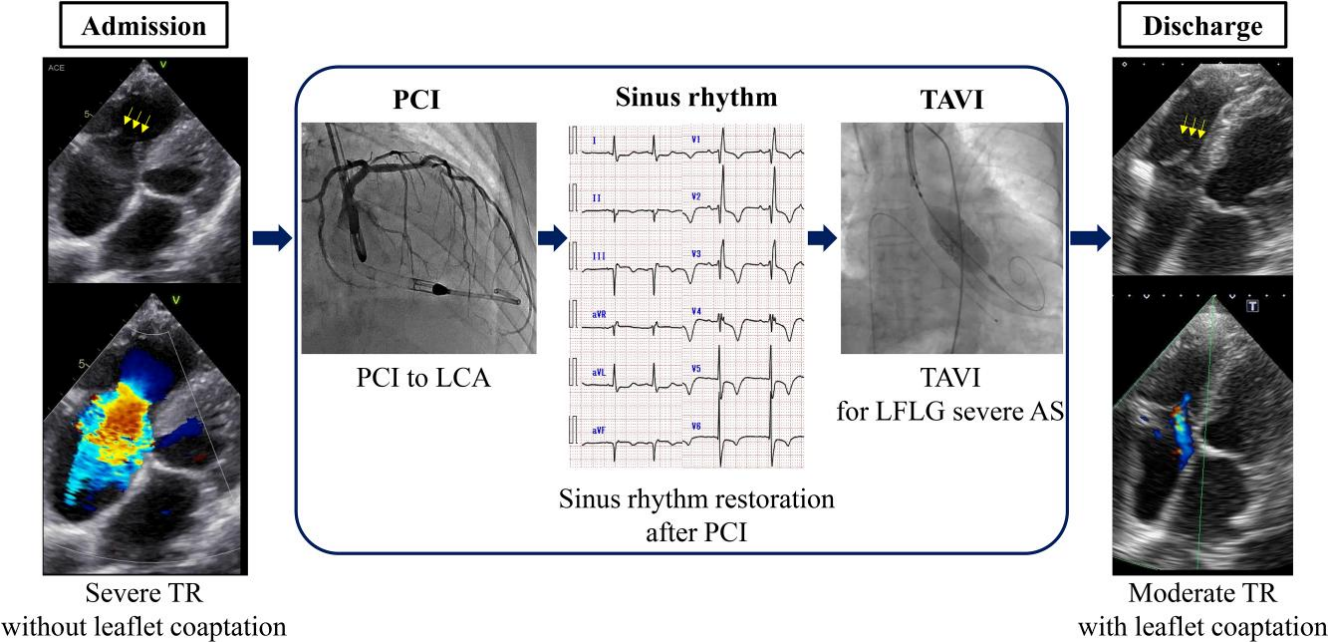


## Case presentation

**Figure 1:**
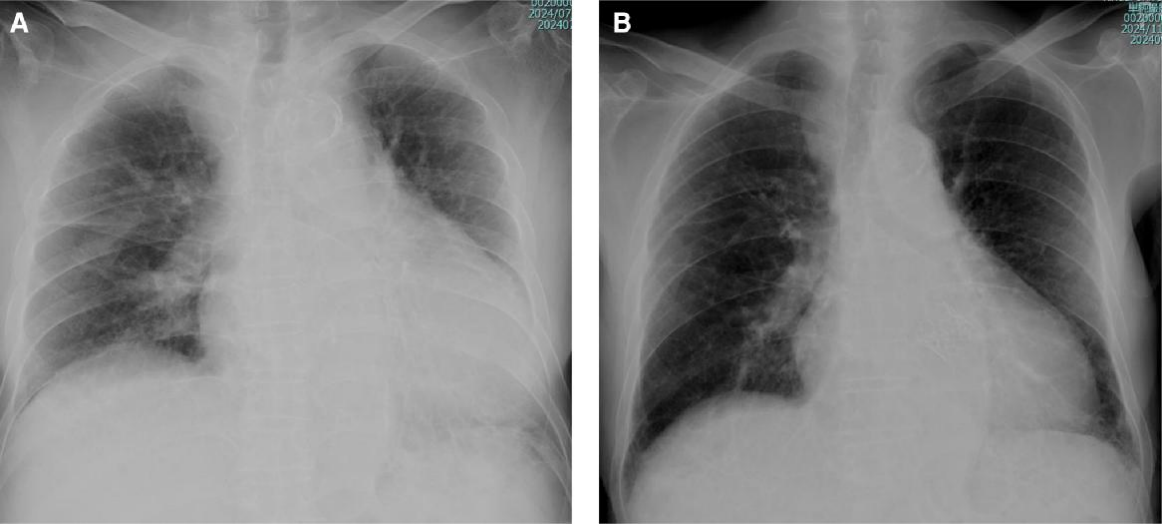
Chest radiography findings. At admission (*A*). At discharge (*B*). Chest radiography at admission demonstrates pulmonary congestion and significant cardiomegaly, both of which are improved at discharge.

**Figure 2:**
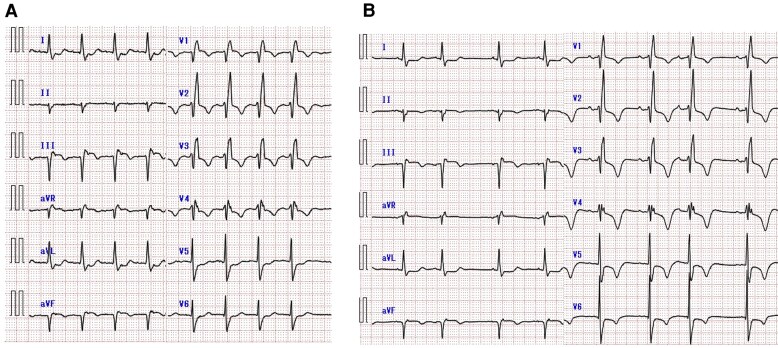
Electrocardiogram findings. At admission (*A*). Following percutaneous coronary intervention (PCI) (*B*). Electrocardiogram at admission shows atrial flutter with a heart rate of 105 bpm. Sinus rhythm is restored after PCI.

**Figure 3:**
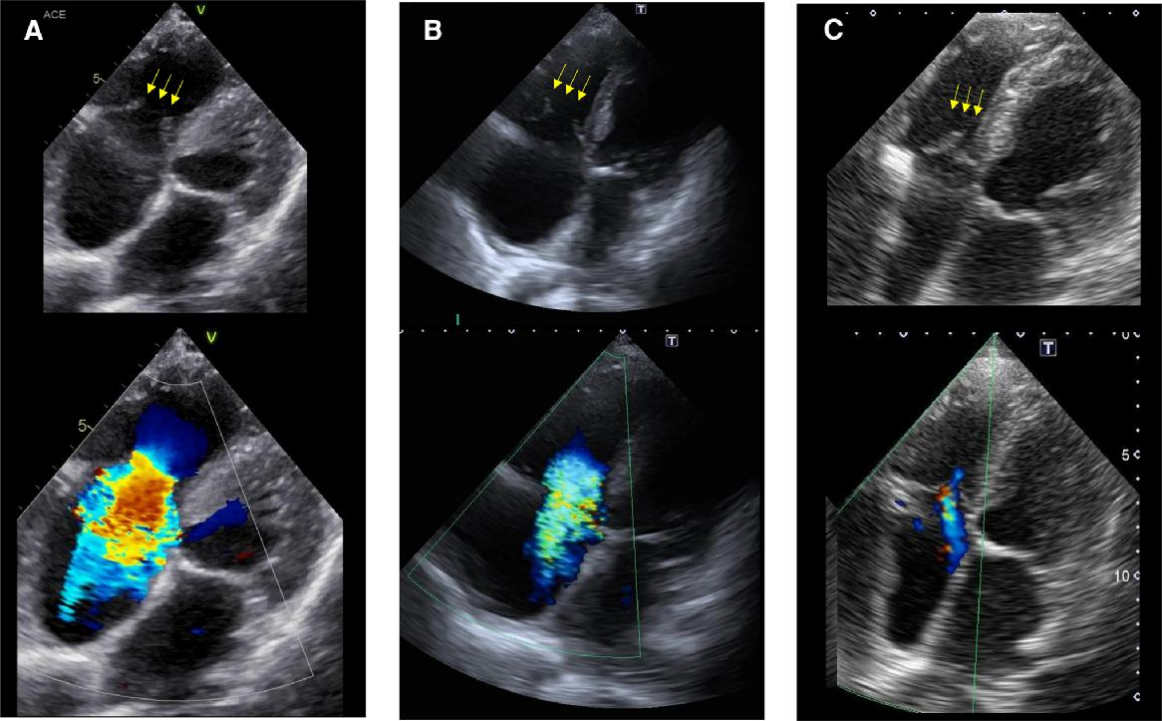
Echocardiographic images showing the progression of tricuspid valve leaflet and tricuspid regurgitation (TR). At admission (*A*). Non-coaptation of the tricuspid valve leaflet (yellow arrows) and severe TR are observed. Percutaneous coronary intervention and sinus rhythm restoration (*B*). Non-coaptation of the tricuspid valve leaflet (yellow arrows) and severe TR are observed. After TAVI (*C*). Coaptation of the tricuspid regurgitation (yellow arrows), right ventricular size reduction, and improvement in TR are observed.

**Figure 4:**
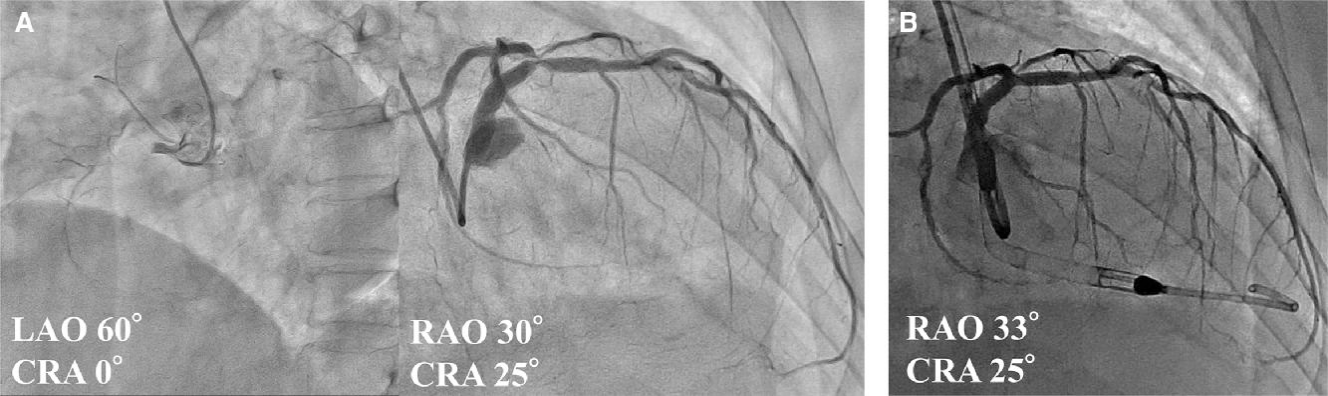
Coronary angiographic findings (*A*). Percutaneous coronary intervention to the left coronary artery (*B*).

A 92-year-old man with a history of hypertension and diabetes mellitus had experienced myocardial infarction approximately 20 years prior. Ten years later, he underwent repeated percutaneous coronary intervention (PCI) for silent myocardial ischaemia. Although cardiac function was initially preserved, it progressively deteriorated, accompanied by aortic stenosis (AS). During the past 2 years, he was repeatedly hospitalized for HF despite medication treatment with empagliflozin 10 mg, bisoprolol 0.3125 mg, and enalapril 5 mg. Although electrical cardioversion was attempted to manage the atrial flutter, the arrhythmia recurred. The patient had exertional dyspnoea at hospital presentation. Physical examination revealed rhonchi in the lungs, a systolic murmur of Levine grade 2/6, and bilateral lower extremity oedema. At rest, the patient’s oxygen saturation was 95% on room air; blood pressure, 109/69 mmHg; and heart rate, 112 beats per minute. Chest radiography revealed cardiomegaly and pulmonary congestion (*Figure 1A*). Electrocardiography demonstrated atrial flutter with a right bundle branch block pattern (*Figure 2A*). Laboratory data showed mildly elevated aspartate aminotransferase levels (42 U/L), along with elevated troponin I (1.390 ng/mL) and NT-proBNP (4570 pg/mL) levels. Based on these findings, the patient was diagnosed with worsening HF and was admitted. Transthoracic echocardiography revealed a left ventricular (LV) ejection fraction of 33% and a stroke volume index of 27 mL/m^2^. Regarding valvular heart disease, an aortic valve peak velocity of 3.3 m/s, a mean pressure gradient of 19 mmHg, and a valve area of 0.44 cm² were observed, all of which were suggestive of low-flow low-gradient AS. Moderate aortic and mitral regurgitations were also observed. Furthermore, the tricuspid valve leaflets did not coapt, resulting in severe TR (*Figure 3A*). Right ventricular (RV) function was impaired, with an RV fractional area change of 22% and a markedly decreased tricuspid annular plane systolic excursion of 6.5 mm. Administration of 20 mg furosemide after admission rapidly improved the HF. Coronary angiography to investigate the aetiology of HF revealed total occlusion of the right coronary artery (RCA) and 90% stenosis from the left main coronary artery to the bifurcation of the left anterior descending and left circumflex arteries. Collateral flow from the left coronary artery (LCA) to the RCA was also observed (*Figure 4A*). Owing to the high surgical risk associated with advanced age and reduced cardiac function, our cardiac team opted for a treatment strategy involving PCI and TAVI rather than conventional surgical treatment. PCI for the LCA was performed before treatment for valvular heart diseases (*Figure 4B*). Meanwhile, PCI for the RCA was not performed due to difficulty in advancing the wire across the RCA, medical therapy was chosen instead. The patient’s cardiac rhythm spontaneously returned to sinus rhythm following PCI (*Figure 2B*). LV ejection fraction improved from 33% to 46%, stroke volume index increased from 27 mL/m² to 30 mL/m², and also mitral regurgitation improved from moderate to mild. However, the severity of AS, TR without valve leaflet coaptation, and symptoms persisted. (*Figure 3B*). Consequently, TAVI with a balloon-expandable valve was performed using the left carotid approach. Remarkably, sufficient coaptation of the tricuspid valve leaflets was observed 1 day after TAVI, along with a reduction in the size of the right atrium and ventricle. This led to moderate improvement in TR severity (*Figure 3C*) and subsequent HF improvement (*Figure 1B*, *Table 1*). At the time of discharge, diuretics were withdrawn, and bisoprolol was up-titrated to 0.625 mg, whereas enalapril had to be discontinued owing to hypotension. The patient did not require rehospitalisation for HF postdischarge.

**Table 1:** Changes in laboratory data and echocardiographic parameters before and after treatment

	At admission	After PCI	After TAVI
***Laboratory data***			
AST, U/L	42	35	34
ALT, U/L	22	16	9
Cre, mg/dL	1.70	1.57	1.10
eGFR, mL/min/1.73m^2	29.7	32.4	47.7
NT-proBNP, pg/dL	4570	2724	2119
***Echocardiographic parameters***			
LVEF, %	33	46	54
LVDd, mm	46	40	43
LVDs, mm	36	31	31
LAD, mm	39	40	43
E wave, cm/sec	96	31	68
Deceleration time, ms	150	194	330
E/e’	14	6	11
MR grade	moderate	mild	mild
RVFAC, %	22	—	45
TAPSE, mm	7	8	18

ALT, alanine aminotransferase; AST, aspartate aminotransferase; Cre, Creatinine; eGFR, estimated glomerular filtration rate; LAD, left atrial dimension; LVDd, left ventricular end-diastolic dimension; LVDs, left ventricular end-systolic dimension; LVEF, left ventricular ejection fraction; MR, mitral regurgitation; NT-proBNP, N-terminal pro-brain natriuretic peptide; RVFAC, right ventricular fractional area change; TAPSE, tricuspid annular plane systolic excursion

## Discussion

This case illustrates that multimodal treatment without direct surgical intervention on the tricuspid valve itself can markedly improve tricuspid valve leaflet coaptation and the severity of TR, highlighting that comprehensive treatment strategies may indirectly improve right-sided valve function and clinical outcome.

The two main subtypes of functional TR are ventricular functional TR (VFTR) and atrial functional TR (AFTR).^1,4^ VFTR is a consequence of dilatation and geometric deformation of the tricuspid annulus resulting from increased RV afterload. It is often linked to progressive left-sided valve, myocardial dysfunction, or pulmonary diseases. In contrast, AFTR is caused by right atrial (RA) enlargement and tricuspid annular dilatation in the context of atrial fibrillation or HF with a preserved ejection fraction.^5^ The current case demonstrated features of both subtypes: RV overload due to coronary artery disease and AS and RA enlargement with atrial flutter. A reduction in LV afterload and pulmonary artery pressure following revascularisation for ischaemic cardiomyopathy and TAVI may improve right heart haemodynamics, and reduce TR severity. TR reduction promotes RV reverse remodelling, and enhance tricuspid valve leaflet coaptation.^6,7^ Furthermore, sinus rhythm restoration improves AFTR by reducing the right atrial size and tricuspid annular dilation.^8^ Catheter ablation therapy for atrial arrhythmias may improve TR.^9^ In the current patient, cardiac rhythm returned to sinus rhythm after myocardial ischaemia treatment. This suggests that the afterload reduction associated with the improvement in myocardial ischaemia contributes to the resolution of the atrial arrhythmia. In addition, a previous study focused on patients with severe AS and concomitant TR revealed that AFTR was associated with the persistence or worsening of TR after TAVI. Moreover, patients with persistent TR had a significantly poorer prognosis than those with improved TR after TAVI.^10^ This suggests that restoring the sinus rhythm may play a key role in managing conditions similar to those in the present case.

In conclusion, the current case shows that comprehensive management of ischaemia, AS, and rhythm control can significantly improve in severe functional TR, even without leaflet coaptation. Individualised multimodal strategies may offer an effective therapeutic pathway for high-risk patients who are ineligible for conventional surgery.

## Data Availability

The data underlying this article are available in the article. The data underlying this article will be shared on reasonable request to the corresponding author. **Consent:** Written informed consent for the publication of this case report and any accompanying images was obtained from the patient, in compliance with COPE guidelines. **Funding:** The authors did not receive any funding for this report.

## Lead author biography

Dr. Minami, MD is an Assistant Professor in the Department of Cardiology at Kindai University, Japan. Her clinical practice covers a wide range of cardiovascular diseases, including heart failure, ischaemic heart disease, arrhythmia, and valvular heart disease. Her research interests include clinical outcomes, imaging, and optimization of medical therapy in cardiovascular medicine. Dr. Minami is actively involved in both patient care and academic research, and she has published several original articles in peer-reviewed journals. She is a member of the Japanese Circulation Society.
